# Books and Authors

**Published:** 1907-11

**Authors:** 


**Progressive Medicine, Vol. III, September, 1907.** A Quarterly Digest of Advances, Discoveries and Improvements in the Medical and Surgical Sciences. Edited by **Hobart Amory Hare,** M.D., Professor of Therapeutics and Materia Medica in the Jefferson Medical College of Philadelphia. Octavo, 290 pages, with 15 engravings. Per annum, in four cloth-bound volumes, $9.00; in paper binding, $6.00, carriage paid to any address. Lea Brothers & Co., Publishers, Philadelphia and New York.

This volume contains much material of special interest to the everyday practitioner. The first article, by William Ewart, London, deals with diseases of the thorax and its viscera, including the heart, lungs, and bloodvessels. It contains some of the latest views on tuberculosis, including the study of dust infection, *x*-ray diagnosis, the opsonic index, the diazo reaction,—another of these illusive methods,—serum agglutination, lung gymnastics, square chest in soldiers, high diaphragm, paralysis of the diaphragm and numerous other topics, giving the recent work in them all.

The section on dermatology and syphilis, by William S. Gottheil, New York, in which the limits of the *x*-ray sphere in general and in dermatology in particular, are pointed out. There are many other points of interest in this article.

Obstetrics is a topic of great importance to every general practitioner, and here, in the article prepared by Edward P. Davis, Philadelphia, will be found many things which may not generally be known but with which it is important to be conversant.

Diseases of the nervous system is the last section of the book, arranged by William G. Spiller, Philadelphia, and contains much of value to the neurologist as well as to the family doctor. Brain tumor is a great topic and in this place some of the symptomatology of this condition is pointed out.

The entire volume, though containing but few illustrations, possesses great merit and unfolds many new or newer points of importance that every physician cannot fail to be interested in.

---

**The Standard Family Physician,** by **Dr. Carl Reissig,** of Hamburg, Germany, and **Dr. Smith Ely Jelliffe,** of New York; 2 volumes; large 8vo; illustrated. New York and London: Funk & Wagnalls Company. 1907. (Buckram binding, $15.00 per set, three-quarters morocco, $20.00, net prices.)

This is the first American attempt to prepare a medical book for family use, by physicians of conceded professional standing. The work is encyclopedic in character, composed of two editorial staffs, American and foreign, each headed by a well and favorably known physician,—Dr. Smith Ely Jelliffe for this country and Dr. Carl Reissig for the European profession. It deals with every disease which it is important for the family to be informed about, and the anatomy of the human body, is sufficiently explained, with the aid of a manikin, to give intelligent direction to the clinical aspects of the text.

One of the purposes, and we hope effects, of the book is to check the sale of patent medicines to the family,—to materially diminish the harm done by these alleged cure-alls that really cure nobody, but which, on the other hand, in many instances create the drug and liquor habits. The sections on hygiene and sanitation cannot fail to produce results of most beneficial character and the entire work is prepared in a manner that must prove helpful to the family doctor, in carrying out his directions regarding the prevention as well as the treatment of disease.

The illustrations are numerous and well adapted to the understanding of the unprofessional reader. The type is large and the heavy side heads make the page attractive; in short, it is difficult to see how a better work, in material or mechanical construction, could have been prepared for the object which it is intended to meet. It seems to occupy its appointed place with a singular fitness of purpose.

---

**Surgery:** Its Principles and Practice. In five volumes. By 66 eminent surgeons. Edited by **W. W. Keen,** M.D., LL.D., Hon. F.R. C.S., Eng. and Edin., Professor of the Principles of Surgery and of Clinical Surgery, Jefferson Medical College, Phila. Volume II. Octavo of 920 pages, with 572 text-illustrations and 9 colored plates. Philadelphia and London: W. B. Saunders Company. 1907. (Cloth, $7.00; half morocco, $8.00 net prices.)

The second volume of this epoch-making work contains thirteen chapters dealing with diseases of the bones, fractures, surgery of the joints, dislocations, surgery of the muscles, tendons and bursæ, orthopedic surgery, surgery of the lymphatic system, surgery of the skin, pathology of the chief surgical disorders of the nervous system, surgery of the nerves, traumatic neurasthenia hysteria and insanity, surgery among the insane, and surgery of the spine. The contributors of these several chapters respectively are Edward Hall Nichols, Daniel N. Eisendrath, Robert W. Lovett and E. H. Nichols, Daniel N. Eisendrath, John Fairbairn Binnie, Robert W. Lovett, Frederick Henry Gerrish, John A. Fordyce, William G. Spiller, George Woolsey, F. X. Dercum, John Chalmers Da Costa, and George Woolsey.

The sections or chapters, the one on fractures and the other on dislocations, by Eisendrath, deserve special comment for their completeness and scientific value. Dercum's chapter on traumatic neurasthenia, traumatic hysteria and traumatic insanity deals with the newer questions of neurosis in surgery, and possesses great scientific interest. The orthopedic section, too, is full of excellent text as well as illustrations.

This second volume is constructed on similar lines as the first, and complements it most generously and satisfactorily. The publishers deserve mention for the splendid manner in which they are producing the work.

**American Practice of Surgery.** A complete System of the Science and Art of Surgery by representative surgeons of the United States and Canada. Edited by **Joseph D. Bryant,** M.D., and **Albert H. Buck,** M.D., New York. Complete in eight volumes. Vol III. Imperial octavo, pp. 775, illustrated. New York: William Wood & Company. 1907. (Price, cloth, $7.00).

Volume three of this colossal work is divided into three parts which are numbered consecutively with the other volumes. Mason (United States Army) deals with poisoned wounds, including the bites and stings of animals and insects; Rambaud (New York City) treats of rabies; Eve (Nashville) deals extensively with fractures; Thomas (Philadelphia) presents the subject of pseudoarthrosis; Peters (Toronto, lately deceased) discusses inflammatory affections of bone; Park (Buffalo) writes on non-inflammatory affections of bones; Horsley (Richmond) treats of syphilitic disease of the bones; Simmons (Boston) handles the subject of tumors originating in bone; Painter (Boston) discourses upon chronic non-tuberculous and non-traumatic inflammations of joints; Primrose (Toronto) offers remarks on the subject of tuberculous disease of the bones and joints; Oliver (Cincinnati) discourses upon wounds of joints, and an elaborate index closes the volume. The book is replete with the best thought on these topics, is excellently illustrated, and covers surgical ground not heretofore occupied. In its entirety this doubtless is the most complete treatise on surgery yet issued or contemplated—provided only that the volumes to be put forth equal those which have been sent out, and of this we feel no question. It is a work that all surgeons will feel proud to possess and which general practitioners will not consult in vain.

---

**International Clinics.** A Quarterly of Illustrated Clinical Lectures and especially prepared articles on Treatment, Medicine, Surgery, Neurology, Pediatrics, Obstetrics, Gynecology, Orthopedics, Pathology, Dermatology, Ophthalmology, Otology, Rhinology, Laryngology, Hygiene and other topics of interest to students and practitioners. Edited by **W. T. Longcope,** M.D. Volume III, seventeenth series. Philadelphia and London: 1907. J. B. Lippincott Co. (Cloth $2.00).

The third volume of the series of 1907, under head of treatment contains—some practical and theoretical considerations concerning diabetes by David L. Edsall; on the treatment of pneumonia and the action of metallic ferments, by Albert Robin; Mechanotherapy, by John W. Wainwright; curability of tuberculosis. The articles under the head of medicine are: blood pressure in tuberculosis, by W. B. Stanton: two cases of primary carcinoma, by William Henry Porter; acute gastritis, by David Somerville: salient factors in the estimation of renal disease, by Raymond Wallace; general abdominal enlargement with reference to hepatic cirrhosis; by Mark A. Brown.

Under the head of surgery the contributions are—inoculability of tumors and the endemic occurrence of cancer, by Leo Loeb; surgery of the bloodvessels, by J. E. Sweet; inflammation of the gallbladder and gall ducts, by George Tully Vaughn; perforating ulcer of the foot, its etiology and treatment, by Arthur J. Hall; some surgical aspects of tuberculosis, by John B. Roberts.

In gynecology two articles appear, one—collargol in puerperal infection, by E. Brownaire; and another—atopomenorrhea, by F. K. Green and T. W. Hunter. The articles on genitourinary diseases are—conservative perineal prostatectomy for chronic prostatitis, by Hugh H. Young; etiology and experimental study of syphilis, by Frederick P. Gay; spermatic insufficiency and diastematic insufficiency. The articles on ophthalmology are—a contribution to the study of ocular birth injury, by Ernest Thompson and Leslie Buchanan; extraction of cataract in the capsule, by J. Rutter Williamson; how to turn back the upper lid, by Raymond Beal.

In neurology there is an article on some cases of polyneuritis, by F. Barkes Weber. In dermatology there is an article on present treatment of hypertrichosis, by L. Brocq. In pathology, an article by Charles F. Craig, on the relation of intracorpuscular conjugation in the malarial plasmodia to latent and recurrent affections, closes the book. The illustrations are numerous and excellent and the entire volume is replete with instructive material.

